# Factors associated with knowledge towards postoperative nausea and vomiting management among health professionals in referral Hospitals of Northwest Ethiopia. A multi-center cross-sectional study

**DOI:** 10.1016/j.amsu.2021.102825

**Published:** 2021-09-08

**Authors:** Yewlsew Fentie Alle, Hailu Yimer Tawuye, Tadesse Belayneh, Abraham Tarekegn Mersha, Tikuneh Yetneberk

**Affiliations:** a Department of Anesthesia, Debre Tabor University, Debre Tabor, Ethiopia; b Department of Anesthesia and Critical Care, College of Medicine and Health Sciences, University of Gondar, Gondar, Ethiopia

**Keywords:** Postoperative nausea and vomiting, Knowledge, Health professionals, Ethiopia

## Abstract

**Background:**

Knowledge of health care professionals on postoperative nausea and vomiting (PONV) and antiemetic prescription trends affects patient's outcome after surgery and anesthesia and also patient and family satisfaction. Hence, knowing the knowledge status of health professionals towards PONV management is vital for the optimal care of surgical patients. Therefore, the study aimed to assess the knowledge and factors associated with PONV management among health professionals in referral hospitals of Northwest Ethiopia.

**Methods:**

An institutional based cross-sectional study was conducted on 407 health care professionals from March 1 to 30, 2019. A Simple random sampling technique was used to select the study participants. Both bivariable and multivariable logistic regression analysis were used to identify factors associated with the knowledge level of health professionals on PONV management. In the multivariable analysis, variables with a p-value <0.05 were considered statistically significant.

**Results:**

In this study, about 52.8% (95% CI: 47.9, 57.2) of the participants had good knowledge of PONV management. Being male (AOR = 1.95; 95% CI: 1.20, 3.17), Physician (AOR = 5.36; 95% CI: 2.20, 13.5), Anesthetist (AOR = 3.88; 95% CI: 1.66, 9.08), and taking training on PONV management (AOR = 5.32; 95%CI: 1.58, 17.89) were positively associated with good knowledgeable of health professionals about PONV management.

**Conclusion:**

and recommendation: More than half of health care professionals who are working in the perioperative sites of the referral hospitals had good knowledge about the PONV management. Being male, Physician, Anesthetist and taking in-service training on PONV management were significantly affecting the knowledge level of health professionals on PONV management. Thus, providing regular in-service training on PONV management, especially for physician and anesthetist is highly recommended.

## Highlights

• Knowledge of health professionals on postoperative nausea and vomiting and antiemetic prescription trends affects patient’s outcome after surgery and anesthesia.
• More than half of the study participants had good knowledge of PONV management.
• Being male, Physician, Anesthetist and taking in-service training on PONV management were significantly affecting the knowledge level of health professionals towards PONV management.

## 1 Introduction

Postoperative nausea and vomiting (PONV) is a common undesirable side effect of surgery and anesthesia, which results in simple to severe complications following surgery [1,2] and also decreases patient and family satisfaction [1]. The incidence of PONV in adults reaches 25–30% and this incidence increases up to 60–80% in high risk patient [3]. A study done in Ethiopia showed that the incidence of PONV was 36.2% [2]. Similarly, a finding from Singapore showed that, 36.5% of physicians had poor knowledge, where as 15.3% respondents had good knowledge and 63.5% had intermediate knowledge [4]The knowledge level of physicians on PONV management as well as antiemetic prescription practices were not well organized that causes PONV still under treated [4].

In the current practices, there was an advancement in the perioperative anesthesia management, antiemetic drugs usage and PONV management strategies, but the PONV incidence is still high [7].

Health professionals should have good knowledge of PONV management and need to implement proper treatment before and after PONV occurrence to improve PONV management trend [10]. However, there are situations in which health professionals can't intervene during PONV, even if the factors causing nausea and vomiting are identified by professionals [5]. This is mainly explained by absence of clearly adopted standardized PONV management protocol [8], poor implementation of knowledge to actual practice [9], limited knowledge of health professionals regarding the consequences of PONV, and most of health professional did not give attention to PONV prevention strategies [6].

Therefore, this study assessed the knowledge level of PONV management and the associated factors among health professionals who are working in referral hospitals of Northwest Ethiopia.

## 2 Materials and method

***Study design*, *period and Area****:* A multi-center cross-sectional study was conducted from March 1 to 30, 2021 in referral hospitals of Northwest Ethiopia. The study was conducted at Debre Markos, Felege Hiwot, Tibebe Gion and University of Gondar Referral Hospitals, which are the biggest hospitals in northwest part of Amhara regional state, Ethiopia.

***Source and study population:*** All Physicians, Anesthetists, Nurses and Midwives that work in the operation room, recovery room and surgical wards of the selected hospitals were source population in this study whereas all physicians, Anesthetists, Nurses and Midwives that work in the operation room, recovery room and surgical wards in the selected referral hospitals during the study period were study populations.

***Sample size and sampling procedure:*** The sample size was determined by using single population proportion formula by considering the following assumptions; 95% confidence interval, 5% margin of error, and 50.6% level of good knowledge [4], and 10% non-response rate. Finally, a sample size of 424 was obtained.

***Sampling technique and procedure:*** Stratified random sampling followed by simple random sampling technique was employed to get the study participants. First of all, health care professionals were stratified into different category's based on their field of study in each hospital. A total number of health care professionals were obtained from human resource management (HRM) of each referral hospital, then total number of health professionals included in the study were proportionated depending on the number of professions in each referral hospital. After all proportional numbers of health professionals were taken from both referral hospitals in each field study, then the simple random sampling technique were employed to select the study participants from each proportioned field of studies. There were a total of 916 health professionals (physicians 365, Anesthetists 93, nurses 254 and midwifes 204) in the selected hospitals during the study period.

### 2.1 Operational definitions

*Knowledge on PONV:* study participants who answered 60% and above correct answers to the knowledge questions on PONV management were considered to have good knowledge, while participants who scored below 60% were considered to have poor knowledge [11,12].

*Post-operative nausea, vomiting management*: Includes components of PONV prevention/risk reduction and postoperative PONV intervention up to post discharge time [13,14].

### 2.2 Data collection procedure

An English version of the self-administered questionnaire was used to collect the data. A total of 25 knowledge questions were used to assess the knowledge level of health professionals towards PONV management. The questioner was taken from a Canada study with some modifications and the reliability coefficient (Cronbach's alpha) was 0.78 [12]. Two anesthetists were assigned in each referral hospital, in which the first one collected data and the other supervises the data collection process. Finally, the sum of correct responses for 25 knowledge questions was computed and expressed by percentage to know whether the study participants have good or poor knowledge.

### 2.3 Data quality assurance

Pretest was done to ensure quality of data in 22 (5% of the sample size) professionals from referral hospitals who were not included in the main study. Then, the necessary corrections were done accordingly to the questionnaire for the main study. A one-day training was given to data collector and supervisor on the aim and objective of the study, approaching of the study participants, the supervision and the data collection process.

The collected data were checked for the completeness, accuracy and clarity. Incomplete data were discarded and counted as non-response. Daily supervision and feedback was given by principal investigator and supervisor during the data collection period.

### 2.4 Data analysis and interpretation

The data were entered, coded and cleaned before statistical analysis. The data were entered by Epidata version 4.2 and exported to SPSS version 20 further analysis. Descriptive statistics were carried out and the results were presented using text, tables and graphs. Both bi-variable and multivariable logistic regression analysis were used to identify factors associated with PONV knowledge of health professionals. Variables with p-value less than <0.2 in the Bivariable logistic analysis was fitted into multivariable logistic regression analysis. Both Crude Odds Ratio (COR) in bivariable logistic regression and Adjusted Odds Ratio (AOR) in multivariable logistic regression with the corresponding 95% Confidence interval were calculated to show the strength of association. In multivariable logistic regression analysis, variables with a p-value of <0.05 was considered as statistically significant.

### 2.5 Registration of research studies

This research is registered with unique identifying number or registration ID: **researchregistry7120.**

Hyperlink to your specific registration (must be publicly accessible and will be checked): https://www.researchregistry.com/browse-the-registry#home/.

In addition, this case series has been reported in line with the STROCSS criteria [15].

## 3 Results

### 3.1 Demographic and work-related characteristics of health professionals

In this study, a total of 407 study participants were involved with the response rate of 96%. The median age of the study participants was 28 (IQR = 26–31) years. The majority of the study participants 278 (68.3%) have less than five years of work experience. In addition, the majority of study participants work in the recovery room and surgical wards **(**Table 1**).**

**Table 1:** Socio-demographic characteristics of health professionals who work in referral hospitals of Northwest Ethiopia, 2019, (n = 407).

Variables	Frequency (n)	Percentage (%)
Age in years		
<25	83	20.4
25-30	222	54.5
31-35	75	18.4
≥36	27	6.6
**Sex**		
Male	272	66.8
Female	135	33.2
**Profession**		
Physician	151	37.1
Anesthetist	43	10.6
Nurse	118	29
Midwife	95	23.3
**Educational level**		
BSc degree	232	57.0
Master's degree	80	19.7
Resident	72	17.7
Specialist and above	23	5.7
**Work experience (years)**		
<5	278	68.3
5-10	92	22.6
>10	37	9.1
**Specific work area**		
Operation room	30	7.4
Recovery room	69	17.0
Surgical wards	193	47.4
Operation and recovery room	115	28.3
**Learn PONV management course in academic class**		
Yes	353	86.7
No	54	13.3
**Take in-service training on PONV management**		
Yes	21	5.2
No	386	94.8

*Surgical wards (general surgery ward, orthopedic surgical ward, gynecology ward, obstetrics surgical ward, ophthalmic wards).

### 3.2 Knowledge level of health professionals about PONV management

**Fig. 1:**
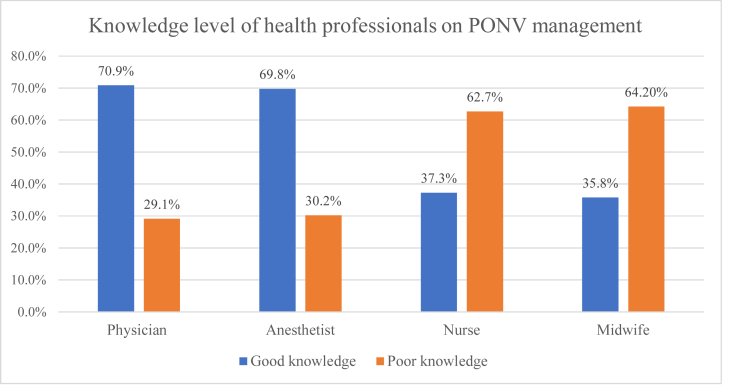
Knowledge level of health professional based on field of study in referral hospitals of north west Ethiopia 2019 (n = 407).

In this study, only 215 (52.8%; (95% CI: 47.9, 57.2)) participants had good knowledge on PONV management. The knowledge level of health professionals varies across professions **(**Fig. 1**).**

In the current study, women are more likely to suffer from PONV than men and metoclopramide can cause drowsiness were the most answered questions, 341 (83.8%), by study participants. On the other hand, the majority of patients are more worried about pain than PONV was the least answered question, 94 (23.1%), by health professionals (Table 2).

**Table 2:** Knowledge questions answered correctly by health professionals who are working in referral hospitals of northwest Ethiopia, 2019, (n = 407).

Knowledge questions (n = 25)	Frequency (n)	Percentage (%)
The overall incidence of PONV is less than ten percent (F)	240	59.0
Women are more likely to suffer from PONV than men (T)	341	83.8
The majority of patients are more worried about pain than PONV (F)	94	23.1
PONV is unpleasant, but rarely causes delay in recovery time after surgery (F)	124	30.5
There is a strong relationship between motion sickness and PONV (T)	283	69.5
Prolonged pre-operative fasting can result in PONV (T)	262	64.4
Use of inhalational anesthetic agents by anesthetists help to reduce the incidence of PONV (F)	169	41.5
Opioids can affect PONV because they increase gastric motility (F)	169	41.5
When transporting back to the ward from recovery room supine position is best for preventing PONV (F)	241	52.6
If there is no evidence of abdominal distention, sips of fluid can usually be recommended 2 h after surgery (T)	205	50.4
Surgery greater than 30 min increase the risk of PONV (T)	303	74.4
Regional anesthesia increases the risk of PONV (F)	198	48.6
Gynecological surgeries are high risk procedures for PONV (T)	281	69
Pediatrics patients age <3 years are higher risk for PONV than older children.	174	42.8
Cimetidine is dopamine antagonist anti-emetic drug (F)	266	65.4
Hypertension is more likely to cause PONV than hypotension (F)	227	55.8
Nausea is a normal reaction to surgery and does not need any intervention unless it results in vomiting (F)	223	54.8
Adequate IV ﬂuid hydration is an effective strategy for reducing the baseline risk for PONV (T)	330	81.1
Smokers are less likely to experience PONV (T)	205	50.4
PONV is more common following orthopedic surgery (F)	204	50.1
TIVA with ketamine is preferred for prevention of PONV (F)	167	41.0
Dexamethasone is considered an effective anti-emetic, especially after laparoscopic surgery (T)	322	79.1
Metoclopramide can cause drowsiness (T)	341	83.8
Acupuncture is a none pharmacological prophylaxis for PONV (T).	327	80.
Promethazine can be administered in any IV site (F)	250	61.4

F = false, T = true.

### 3.3 Factors associated with knowledge of health professionals on PONV management

In the bivariable logistic regression analysis, gender, profession, level of education, learning PONV management in academic classes and training on PONV management were significant. However, only gender, profession and taking in-service training on PONV management were significantly associated with good knowledge.

The multivariable logistic regression analysis showed that the odds of having good knowledge among males were 1.95 times (AOR = 1.95; 95% CI: 1.20, 3.17) higher compared to females. Similarly, the odds of having good knowledge towards PONV management was 5.36 times (AOR = 5.36; 95% CI: 2.20, 13.05) higher among physicians and 3.88 times (AOR = 3.88; 95% CI: 1.66, 9.08) compared with midwives. Finally, the likelihood of having good knowledge towards PONV management was 5.32 times (AOR = 5.32; 95% CI: 1.58, 17.89) higher among health professionals who had taken PONV management in-service training as compared with professionals who didn't take training (Table 3).

**Table 3:** Factors affecting the knowledge level of health professionals working in referral hospitals in northwest Ethiopia (n = 407).

Variables	Knowledge level		Crude OR (95% CI)	Adjusted OR (95% CI)	p-value
	Good, (n%)	Poor, (n%)			
Sex					
Male	166(77.2%)	106(55.2%)	2.75(1.79, 4.21)	1.95 (1.20, 3.17)	0.007*
Female	49(22.8%)	86(44.8%)	1.00	1.00	
**Profession**					
Physician	107(49.8%)	44(22.9%)	0.07(0.02,0 .30)	5.36(2.20, 13.05)	0.001*
Anesthetist	30(14.0%)	13(6.8%)	0.19(0.04, 0.86)	3.88(1.66, 9.08)	0.002*
Nurse	44(20.5%)	74(38.5%)	0.15(0.03, 0.69)	1.06(0.58, 1.94)	0.85
Midwife	34(15.7%)	61(31.8%)	1.00	1.00	
**Level of education**					
BSc degree	97(45.1%)	135(70.3%)	1.00	1.00	
Master's degree	53(24.7%)	27(14.1%)	2.73(1.61, 4.65)	0.87(0.41, 1.82)	0.71
Resident	44(20.5%)	28(14.6%)	2.19(1.27, 3.76)	0.50(0.20, 1.26)	0.14
Specialist & above	21(9.8%)	2(1.0%)	14.61(3.35,63.80)	3.10(0.59, 16.27)	0.18
**learned PONV in academic classes**					
Yes	194(90.2%)	159(82.8%)	1.92(1.07, 3.45)	0.86(0.45, 1.66)	0.65
No	21(9.8%)	33(17.2%)	1.00	1.00	
**Took training**					
Yes	17(7.9%)	4(2.1%)	4.04(1.33,12.21)	5.32 (1.58, 17.89)	0.007*
No	198(92.1%)	188(97.9%)	1.00	1.00	

* = p-value <0.05, 1 = reference, Crude OR = crude odds ratio, Adjusted OR = Adjusted odds ratio.

## 4 Discussion

Knowledge of health professionals on PONV management approaches have a great role in the reduction of PONV following anesthesia and surgery whereas limitations in knowledge of health professionals leads to unnecessary adverse effects of PONV, which has the most undesirable outcome to patients [4].

This study shows that, the knowledge level of health professionals on PONV was 52.8% (95% CI:47.9–57.2). This result is relatively similar to a study done in Singapore pediatrics surgeons, in which 50.6% of respondents had good knowledge in PONV management and identifying risk factors in pediatric patients [4]. This similarity might be supported by the reason that most of the physicians had closely related work experience with the current study.

The mean percentage score correctly answered in this study was 59.6%, which is similar with a study done in Canada with a mean percentage score of 61.34% [12]. This might be due to the reason that both studies use the same PONV management knowledge assessment tool.

In this study, male participants were more knowledgeable than females. However, a study done in USA on knowledge of certified registered nurse anesthetists showed that females had a higher knowledge on PONV management than males (mean score of 45.82 vs 43.21) [16]. The possible explanation for this variation might be due to the difference in the knowledge assessment tools, type of profession and level of education since this study included a variety of professions with different level of education.

In addition, the current study revealed that physicians and anesthetists were more knowledgeable than Midwives. This might be due to the nature and scope of the professions that they acquire knowledge on PONV management and the potential exposure to patients who had repeated PONV in their working area [17]. In addition, most of the physicians and anesthetists evaluate patients, order prescription and administer PONV prophylaxis for surgical operative patients with risk of perioperative nausea and vomiting in wards, recover room and operation theatre.

Training on PONV management was significantly associated with the knowledge level of health professionals on PONV management. Study participants who had taken training were more knowledgeable than their counterparts. This might be due to health professionals who had taken PONV management training could improve the baseline knowledge and acquire updated information about PONV management protocols which results no fad in knowledge [18].

Prevention of PONV by giving prophylactic antiemetic drugs and early management when PONV happens are vital interventions of health professionals in reduction of PONV [19]. However, lack of standard protocol for prevention and management of PONV and also variations in availability of antiemetic drugs affects the prevention and management of patients with repeated PONV [20].

In the current study, the most commonly used drugs for postoperative nausea and vomiting dexamethasone, cimetidine and metoclopramide. On the contrary, 5-HT3 antagonists were the commonly used medications in study done on USA [19]. In fact, this difference might be those drugs are not available in the study areas (Ethiopia) even the cost of drugs might not be affordable.

In this study, as a limitation, there was no standardized knowledge assessment tool that includes all health professionals, since the current tool was taken from a modification of the nurse's knowledge assessment tool. In addition, there might be a recall baize in the assessment tools due to individuals' performance difference. On the other hand, there were a limited number of studies done in this area to compare the current study results.

## 5 Conclusion

In this study, more than half of health professionals had good knowledge of PONV management. Gender, profession, and participants who took training on PONV management were significantly associated with the knowledge level of health professionals. Hence, health professionals should have to get regular training/seminar on PONV management and increase professional development programs.

## Declaration of competing interest

The authors declare that there is no conflict of interest.

## Data Availability

The dataset is available upon request from the corresponding author due to ethical restrictions and privacy concerns from Abraham Tarekegn: abrahamtm2006@gmail.com.

## Abbreviations

AOR: Adjusted Odds Ratio
COR: Crude Odds Ratio
HRM: Human Resource Management
5-HT3: 5-HydroxyTryptamine-3
PONV: Post Operative Nausea Vomiting
TIVA: Total Intra-Venous Anesthesia
UoG: University of Gondar
USA: United States of America

## Registration of research studies

1. Name of the registry:

2. Unique Identifying number or registration ID:

3. Hyperlink to your specific registration (must be publicly accessible and will be checked):
